# Expression of an intron-containing gene from *P. falciparum* using three sequential PCR steps

**DOI:** 10.6026/973206300212278

**Published:** 2025-08-31

**Authors:** Ameer Abbas, Manoj Yadav, Vikas Kumar, Ankit Gupta

**Affiliations:** 1 Department of Biochemistry, All India Institute of Medical Sciences, Raebareli, Uttar Pradesh, India

**Keywords:** Intron, Exon, cDNA, gene expression, PCR amplification, *P. falciparum*

## Abstract

The amplification of eukaryotic genes, with introns is very tedious and expensive. The only available lab method is through cDNA synthesis, which has a very low success rate in generating a pure gene fragment. Therefore, it is of interest to devise a strategy to amplify a *P. falciparum* RPL12_mito_ gene (PfRPL12_mito_) containing an intron through three sequential PCR steps using genomic DNA (gDNA) and primers with a minimum 15 bp 5'-overhang. The absence of an intronic segment and a continuous exonic region in the amplified gene fragment was confirmed using Sanger sequencing.

## Background:

Splicing is a key step during the expression of eukaryotic genes containing introns which encompass the removal of introns and joining of adjacent exons [1]. *In vitro* expression of gene containing introns involves the synthesis of cDNA from RNA through reverse transcription using oligo (dT) primers [2]. The complete removal of genomic DNA (gDNA) contamination from cDNA before PCR amplification is a prerequisite for either recombinant protein expression or protein overexpression [3]. Mild DNase treatment of cDNA often leaves trace amounts of genomic DNA, leading to the unintended amplification of gene fragments containing intronic regions [4]. In contrast, harsh DNase treatment compromises cDNA quality, resulting in little to no amplification of intron-free gene fragments [5]. Therefore, the amplification of gene fragments without introns from cDNA is mostly expensive, tedious, exhaustive, and unfeasible [6]. The alternative ways are either outsourced gene or dsDNA (G-blocks) synthesis, but both are expensive and time-consuming approaches [7]. Therefore, it is of interest to develop a method to amplify a gene fragment lacking an intron without cDNA synthesis.

## Materials and Methods:

## Parasite culture:

*P. falciparum* (3D7) parasites were cultivated at 37°C under 5% O_2_, 5% CO_2_, 90% N_2_ in O^+^ human erythrocytes, RPMI 1640 (Invitrogen) supplemented with 25 mM HEPES, 50 µg/ml hypoxanthine, 0.5% albumax (Gibco), and 0.23% NaHCO_3_. phenol-chloroform method.

## Primer design, gDNA isolation and PCR amplification:

The reference sequence of *Plasmodium falciparum* RPL12_mito_ gene (PF3D7_0212200; PfRPL12_mito_) was retrieved from PlasmoDB site (http://PlasmoDB.org) which contained a single intron (147bp) towards 3'-end followed by exon2 (71 bp). The primers were designed based on vector map (SnapGene) of the pET28a vector with the PfRPL12_mito_ sequence containing an intron. The strategy was devised to amplify the full-length PfRPL12_mito_ gene fragment (765 bp) without an intron through three sequential PCR steps without synthesizing cDNA (Figure 1 - see PDF). Four sequence-specific primers were developed and designated as p1, p2, p3, and p4, which contained 5-overhang complementary to the vector or PfRPL12_mito_ gene sequence as given in Table 1 (see PDF). The length of primers p1, p2, and p3 was 45 bp; whereas, the p4 primer was 50 bp long. Genomic DNA (gDNA) was isolated from *In vitro* blood-stage *P. falciparum* (3D7) parasites (predominantly in the trophozoite stage) using the Quick-DNA MiniPrep kit (ZYMO Research) and used as a template for PCR1-3 as a reference (Figure 2a - see PDF). PCR1 amplification (736 bp) was done with primers (p1 and p2) using parasite gDNA (10ng/reaction) and SpeedSTAR HS DNA polymerase (Takara) at an annealing temperature of 62.4°C. The PCR product was purified by NucleoSpin PCR clean-up kit (Macherey-Nagel) and used as template for PCR2 (769 bp) with primers (p1 and p3) at an annealing temperature of 60.2°C. At last, PCR3 (807 bp) was done with purified PCR2 as a template using primers (p1 and p4) at an annealing temperature of 62.4°C (Figure 2b - see PDF).

## Molecular cloning and recombinant protein expression:

PCR3 was cloned in the pET28a expression vector at BamHI and XhoI sites using the HD Infusion cloning kit (Takara), and clones were confirmed through restriction digestion with the same restriction enzymes, yielding bands of ~5.3kb and 771bp (Figure 2c - see PDF). The sequence of PfRPL12_mito_ encompassing exons 1 and 2, and lacking the intronic segment in plasmid clones was further confirmed by Sanger sequencing (Figure 2d - see PDF). The plasmid clone was co-transformed with the RIG plasmid into BL21 (DE3) cells to express recombinant PfRPL12_mito_ encompassing both n- and c-terminal 6xHis tag, respectively. The expression of recombinant PfRPL12_mito_ protein (~35 kDa) was confirmed by western blotting using anti-His tag antibody (Sigma; 1:1000 dilution) and 3, 3-Diaminobenzidine (Sigma) (Figure 3 - see PDF).

## Results:

Our method strategy bypasses tedious and expensive steps of synthesizing RNA, followed by cDNA synthesis. In most cases, cDNA either retains genomic DNA contamination despite DNase treatment or undergoes degradation with rigorous DNase treatment; both scenarios lead to poor or no amplification of the desired intron-free gene fragment. Consequently, cDNA-based gene amplification often fails or requires multiple attempts to achieve successful protein expression. Alternative approaches, such as dsDNA cloning or gene synthesis, are typically expensive and time-consuming. Here, a method has been devised to PCR-amplify intron-containing genes directly from eukaryotic genomes, such as *Plasmodium falciparum*, for protein expression purposes. We successfully amplified the PfRPL12_mito_ gene fragment encompassing only exons and no intron through three sequential PCR steps with primers containing 5'overhang (Figure 1 - see PDF). Primer p2 was designed in such a way that it contained 3' ends (30 bp) homologous to exon 1 and a 5' overhang (15 bp) homologous to the exon 2 segment. PCR1 was the key step for this strategy to PCR amplify a gene fragment lacking an intronic segment, which was further used as a template for PCR2 (Figure 2a-b - see PDF). Thereafter, PCR2 was used as template to amplify the full-length PfRPL12_mito_ gene (PCR3), which was cloned in the pET28a expression vector at BamHI and XhoI sites (Figure 2c - see PDF). Sanger sequencing of the resulting full-length PfRPL12_mito_ gene fragment confirmed the lack of intron and presence of exons 1 and 2, both in frame and in continuation (Figure 2d - see PDF). Furthermore, the western blotting confirmed the successful expression of full-length PfRPL12_mito_ protein with a 6xHis tag (Figure 3 - see PDF).

## Discussion:

Traditional approaches for expressing eukaryotic genes involve the isolation of RNA followed by reverse transcription to cDNA. However, these steps are not only labour-intensive and costly but also suffer from technical limitations. A significant concern is the persistent contamination of cDNA with genomic DNA despite DNase treatment, or conversely, the degradation of cDNA under harsh DNase conditions. Both outcomes hinder the amplification of clean, intron-free gene fragments required for accurate protein expression. Additionally, alternatives such as dsDNA cloning or commercial gene synthesis, although effective, are resource-intensive and not always accessible, especially in resource-limited settings. To address these challenges, we developed a streamlined method for direct PCR amplification of intron-free gene fragments from eukaryotic genomes, *e.g.* *Plasmodium falciparum*. A key aspect of this strategy is the use of sequential PCR steps with primers designed to bridge exon-exon junctions. Specifically, the second primer (p2) incorporates a 3' region homologous to exon 1 and a 5' overhang homologous to exon 2, facilitating the seamless exclusion of intronic regions during amplification. This eliminates the need for RNA processing and ensures that only exonic sequences are retained in the final product. Furthermore, the successful amplification of the PfRPL12_mito_ gene fragment comprising only exons was validated through Sanger sequencing. The sequence continuity and correct frame alignment of exons 1 and 2 demonstrate the accuracy of the amplification strategy. Importantly, western blot analysis verified the expression of full-length PfRPL12_mito_ protein tagged with 6xHis, further substantiating the functional relevance of this approach. This method presents several key advantages: (i) it bypasses RNA extraction and reverses transcription, (ii) avoids the risk of intron retention or incomplete splicing, (iii) is more economical and time-efficient than gene synthesis and (iv) is broadly applicable to other eukaryotic genes with known exon-intron boundaries. It is especially valuable for organisms with high AT-rich content or where RNA is difficult to isolate, such as *Plasmodium falciparum*. Overall, the method provides a robust and accessible alternative for gene amplification and expression studies.

## Conclusion:

We describe an efficient PCR-based method to amplify and express eukaryotic genes containing introns without relying on RNA extraction or cDNA synthesis. By circumventing the challenges associated with genomic DNA contamination and cDNA degradation, our strategy offers a cost-effective, time-saving, and reliable alternative to conventional approaches. The successful amplification, sequencing, and expression of the intron-free PfRPL12_mito_ gene fragment from the *Plasmodium falciparum* genome validated the utility and robustness of this approach. However, the strategy has certain limitations, particularly its applicability only to genes containing a single intron.

## Abbreviations:

cDNA - Complementary DNA

gDNA - Genomic DNA

RNA - Ribonucleic acid

PCR - Polymerase chain reaction

RPL - Ribosomal Protein Large Subunit

Pf - *Plasmodium falciparum*

## Ethics approval:

The study includes the work which was duly approved by both the Institutional Ethics Committee (2024 -1- EMP- 8) and the Institutional Biosafety Committee (IBSC-2024-1-EMP-03).

## Funding Statement:

The work is funded by a project grant (CST/D-896) received from the Council of Science and Technology (CST), Uttar Pradesh.

## Consent to publish:

Not applicable

## Data availability:

All relevant data are within the manuscript and its Supporting Information files.
